# Role of GPER-Mediated Signaling in Testicular Functions and Tumorigenesis

**DOI:** 10.3390/cells9092115

**Published:** 2020-09-17

**Authors:** Adele Chimento, Arianna De Luca, Marta Claudia Nocito, Paola Avena, Davide La Padula, Lucia Zavaglia, Vincenzo Pezzi

**Affiliations:** Department of Pharmacy, Health and Nutritional Sciences, 87036 Arcavacata di Rende Cosenza, Italy; ariannadl@hotmail.it (A.D.L.); nocitomarta90@tiscali.it (M.C.N.); paox1982@hotmail.it (P.A.); davidelapadula@live.it (D.L.P.); luciazavaglia@hotmail.it (L.Z.)

**Keywords:** GPER, testis, germ cells, Leydig cells, Sertoli cells, telocytes, testis physiology, testicular cancer

## Abstract

Estrogen signaling plays important roles in testicular functions and tumorigenesis. Fifteen years ago, it was discovered that a member of the G protein-coupled receptor family, GPR30, which binds also with high affinity to estradiol and is responsible, in part, for the rapid non-genomic actions of estrogens. GPR30, renamed as GPER, was detected in several tissues including germ cells (spermatogonia, spermatocytes, spermatids) and somatic cells (Sertoli and Leydig cells). In our previous review published in 2014, we summarized studies that evidenced a role of GPER signaling in mediating estrogen action during spermatogenesis and testis development. In addition, we evidenced that GPER seems to be involved in modulating estrogen-dependent testicular cancer cell growth; however, the effects on cell survival and proliferation depend on specific cell type. In this review, we update the knowledge obtained in the last years on GPER roles in regulating physiological functions of testicular cells and its involvement in neoplastic transformation of both germ and somatic cells. In particular, we will focus our attention on crosstalk among GPER signaling, classical estrogen receptors and other nuclear receptors involved in testis physiology regulation.

## 1. Introduction

The mammalian testis is divided into two compartments, the seminiferous tubules including germ cells in various development stages (spermatogonia, spermatocytes, spermatids, spermatozoa) supported by Sertoli cells and the interstitial tissue consisting of loose connective tissue, blood and lymphatic vessels, Leydig cells, fibroblasts, macrophages, leukocytes, and telocytes [1,2]. Testis physiological function consists of spermatogenesis, a process leading to gametes formation occuring in seminiferous tubules regulated by autocrine/paracrine factors, and steroidogenesis that occurs in Leydig cells [3]. Normal male reproductive development and function are controlled by a complex endocrine regulation in which a proper balance between androgens and estrogens plays a pivotal role [4,5].

Cellular response to estrogens is mediated through interaction with nuclear ERs α and β, which activates genomic and non-genomic signaling [6,7,8,9,10,11]. In the genomic pathway, the estrogens/ERs complex, binding ERE either directly or indirectly via transcription factors, modulates gene expression in many tissues, including those of the male reproductive tract [7,12,13]. In addition to the classical model of signal transduction, non-genomic mechanisms have been identified for estrogens and provide that their biological effects do not only arise from direct or indirect interaction of ERs with DNA [8,9,10]. It has also been reported that ERs and their splicing variants are localized to plasma membrane-mediating non-genomic signaling [10,14,15,16]. Moreover, several studies revealed that estrogens act also through GPER [17,18]. GPER, originally described as orphan receptor GPR30, is a member of GPCR cell-membrane proteins superfamily, which have a binding domain inside the plasma membrane and endoplasmic reticulum [17].

Estradiol binds to GPER with a high affinity while estrone and estriol have very low binding affinities [17,19]. Furthermore, several environmental estrogens bind to GPER and activate the downstream signaling pathways, such as BPA, genistein, and nonylphenol [20]. A synthetic specific ligand of GPER, G1 [21], together with G15, a specific antagonist, are used as a target tool to evaluate the GPER function in different cells and disease models [22]. GPER is able to mediate both genomic and non-genomic response with its ligands in both normal and cancer cells [23,24,25,26,27]. Particularly, GPER activation determines multiple intracellular events such as EGFR transactivation leading to rapid ERK1/2 activation, PLC and PI3K phosphorylation, AC stimulation, and intracellular calcium mobilization [17,23,25,26,28,29].

It has been well established that GPER is expressed in testicular cells where it regulates specific functions [30,31,32,33], but it can also be involved in pathological processes, such as cancer [27,34], including estrogen-dependent testicular tumors [35]. In our previous review [35], we pointed out a role of GPER in mediating estrogen action during spermatogenesis and testis development. In addition, we evidenced that GPER seems to be involved in modulating estrogen-dependent testicular cancer cell growth; however, the effects on cell survival and proliferation depend on specific cell type.

There is a controversy whether GPER acts as an autonomous estrogen receptor or whether GPER interacts with nuclear estrogen receptor signaling pathways in response to estrogens or whether it co-operates with other receptors [36]. Studies performed on knockout mice and cultured cells suggest that GPER can act as an autonomous receptor and can also interact with nuclear estrogen receptors. However, the degree to which GPER acts autonomously likely depends on cell type, differentiation status and pathology [i.e., whether the cell is quiescent, proliferative or cancerous] [36]. The more severe testicular phenotype of ArKO mice, compared ERKO mice, supports the hypothesis that an alternative receptor [that could be GPER] and alternative pathways could be involved in mediating estrogen effects on spermatogenesis. Thus, the generation of a triple KO [ESRs and GPER] would be useful to highlight the cross-talk and functional redundancy between the three different receptors as well as between genomic and non-genomic effects exerted by estrogens in the modulation of spermatogenesis and testicular tumorigenesis [35].

In this review, we update the knowledge obtained in the last years on GPER roles in regulating physiological functions of testicular cells and its involvement in neoplastic transformation of both germ and somatic cells. In particular, we will focus our attention on crosstalk among GPER signaling, classical estrogen receptors and other nuclear receptors involved in the testis physiology regulation.

## 2. GPER Role in Testicular Interstitial Compartment

**Figure 1:**
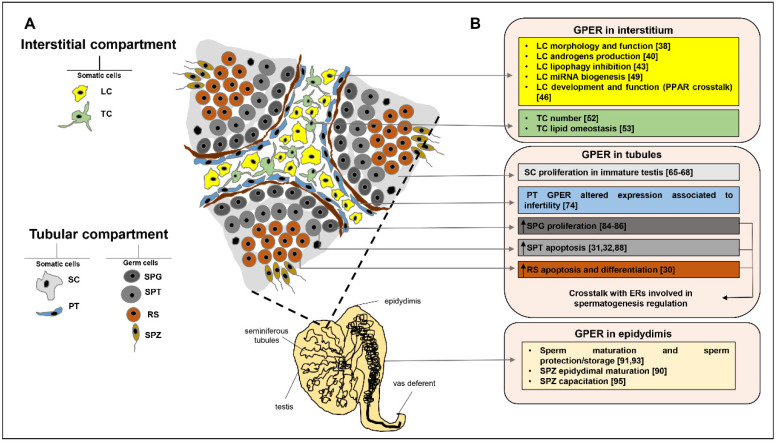
GPER role in testicular interstitial and tubular compartment and in epididymis. In this figure are summarized the knowledge of GPER role (**B**) in testicular interstitial and tubular compartments (**A**) and in epididymis. The drawing represents a cross section of seminiferous tubules, surrounding interstitium and several testicular cell types (**A**). LC: Leydig cells; TC: telocytes; SC: Sertoli cells; PT: peritubular cells; SPG: spermatogonia; SPT: spermatocytes; RS: round spermatids; SPZ: spermatozoa.

Testicular interstitial compartment, located between seminiferous tubules, is delimited from them by a layer of peritubular-myoid cells. Multiple interactions between all testicular cell types and mediated by paracrine factors, influence either cellular growth, differentiation or death [37]. Several studies have investigated GPER role in this intricate network, regulating testicular functions. In this section, we will summarize the results obtained in studies focused on GPER role in Leydig cells and in telocytes (Figure 1).

### 2.1. GPER in Leydig Cells

Interstitial Leydig cells are responsible for testis steroidogenesis [1]. Kotula-Balak and colleagues described for the first time that GPER is able to influence Leydig cell morphology and function [38]. In this study, effects of GPER antagonist G15 were evaluated on both C57BL/6 mice at different ages (immature, mature and aged) and on mouse MA-10 Leydig tumor cell line. The authors demonstrated that after G15 treatment, no effect on immature testis was observed, while an overgrowth of interstitial tissue was found in both mature and aged testes. Alterations in the structure and distribution of various Leydig cell organelles after GPER blockage respect to control, were also observed. Immunohistochemical analysis revealed the presence of more lipid droplets in immature Leydig cells, large mitochondria and numerous lipid droplets in the mature ones, and concentric structure of endoplasmic reticulum between normal-looking and normal-distributed mitochondria in aged cells. Moreover, the use of G15 determined a marked decrease in activity of mitochondria and cytoskeleton, cellular structures directly involved in steroid hormone production [39]. In addition, in both in vivo mice of different ages and in in vitro Leydig cells, G15 treatment determined an increase in ERα and ERβ and aromatase mRNA expression. However, after GPER blockage, intratesticular androgen concentration significantly increased in immature mice, decreased in mature males and did not change in aged animals. These observed changes after G15 treatment reflected Leydig cell heterogeneity to estrogen regulation during male life.

Another study demonstrated that GPER activation, using both estradiol [E2] and selective agonist G1, decreased in a dose-dependent manner, testosterone production in rat Leydig primary cultures. The observation that E2 and G1 determined the same decrease in testosterone production also in adult human testis, suggested that GPER-dependent non genomic signaling represented an important mechanism regulating estradiol-dependent steroidogenesis in human testis [40].

Studies performed on GPER knockout mice revealed GPER involvement in the regulation of obesity, insulin resistance and glucose intolerance [41]. In particular, a relationship between GPER activity and lipid metabolism derived from observation that GPER knockout mice developed visceral obesity and showed an increased level of low density lipoproteins [41]. Milon and collegues demonstrated that in mouse MA-10, tumor cells resembling the immature type of adult Leydig cell lineage [42], lipid homeostasis and metabolism were affected by estrogens [43]. This study demonstrated that G15 induced protein expression changes of steroidogenic [LHR and 3β-HSD] and lipid droplet [PLIN and LC3] markers. Specifically, LHR, 3β-HSD, PLIN, and LC3 expression decreased, while degenerating lipid droplets appeared, indicating lipophagosome formation [43]. These results suggested a GPER role in lipophagy inhibition that is a crucial event in maintaining lipid homeostasis [44] and testosterone biosynthesis.

In this context, the functional interaction among GPER and pathways activated by different receptors is an interesting aspect that was recently studied in Leydig cells. Gorowska–Wojtowicz and colleagues [45] for the first time have shown the importance of relationship between GPER-PPARα and PPARγ in maintaining morphological and functional state of Leydig cells. GPER is an important partner of PPARα and PPARγ in steroidogenic state regulation of Leydig cells through both direct and indirect control at different regulatory levels. In particular, GPER and PPARα action occurs through the PI3K/Akt pathways, while PPARγ prefers the Ras/Raf pathways. Moreover, modified GPER-PPAR crosstalk was found in human LCTs, being a possible cause of LCTs development. This last aspect will be explained in the paragraph on GPER role in testicular tumors. A very recent study by Kotula–Balak and colleagues [46] asserted that exists a GPER-ERRβ-PPARγ interaction in the immature wild boar testicle that affects Leydig cells function [46]. It also highlights the involvement of these receptors in cellular processes through cAMP activation and Raf/Ras/ERK pathways modulating cholesterol concentration and estradiol levels. These cellular and molecular regulations seem to be crucial for the proper development and functions of Leydig cells.

Another interesting aspect recently evaluated is the GPER involvement in miRNA-estrogen regulation [47]. Dysregulation of testicular steroidogenic function by BPA through its action on miRNA has been demonstrated in Leydig cells isolated from murine testis [48]. Recently, Pawlicki and colleagues, using G15, xenoestrogen BPA and its derivatives TBBPA and TCBPA, clarified the GPER involvement on epigenetic regulation in immature boar Leydig cells [49]. The authors suggested that both G15 and different xenoestrogens, except for BPA and G15 plus TCBPA, reduced the GPER protein expression. Furthermore, the use of the above mentioned chemical compounds, modulated the expression of some proteins that are important for miRNA biosynthesis in Leydig cells. In particular, the EXPO5 and DICER mRNA expressions were downregulated while the DROSHA and AGO2 mRNA expressions were significantly upregulated only by G15 plus BPA and TCBPA, respectively. These results confirmed that GPER-modulated expression exerted by G15 and BPA derivatives affected the levels of proteins controlling miRNA biogenesis and function.

### 2.2. GPER in Telocytes

Recent studies, confirmed the presence of telocytes in interstitium; these cells are a novel type of interstitial cell, named also interstitial Cajal-like cells that are involved in testicular homeostasis maintenance and spermatogenesis regulation [50]. Telocytes are easily distinguished from other interstitial tissue cells for the presence of telopodes [51], a long prolongation containing caveolae, mitochondria and endoplasmic reticulum. Recently, Pawlicki and colleagues confirmed telocytes presence in mouse testis and investigated the GPER role in this cell type [52]. Interestingly, the same authors, inactivating GPER trough G15, demonstrated protein expression changes of telocytes functional markers such as CD34, c-kit, PDGFRα and β, VEGF, and vimentin. Moreover, GPER inhibition caused an increase in telocytes number, ERRs mRNA expression and mouse testis relaxin concentration, a protein exclusively secreted by Leydig cells. These results suggested that telocytes number and the expression of important proteins regulating interstitial compartment physiology may be modulated by GPER activity.

Telocytes presence has been demonstrated in testicular interstitium of bank voles [53]. It is known that in these photoperiodic rodents, melatonin endogenously controls reproductive system function through interaction with the hypothalamic–pituitary–gonadal axis. Recently, it has been demonstrated that in male bank voles bred under different light cycles [long day, LD; short day, SD], the photoperiod and melatonin signaling regulate telocytes distribution [53]. Surprisingly, melatonin concentration in these animals is regulated by GPER, decreasing in both LD and SD animals after G15 treatment. In addition, the same authors highlighted that GPER signaling regulated telocyte marker CD34 expression and it is implicated in lipid metabolism. In fact, in GPER-blocked testis, single telocytes were present in the interstitium of LD animals, while in that of SD animals they were absent. Concomitantly, in bank vole interstitial tissue, GPER inhibition induced a decrease in leptin and adiponectin expression and an increase in cholesterol content, suggesting a possible role in maintaining lipid balance and steroidogenic efficiency of interstitial tissue.

## 3. GPER Role in Testicular Tubular Compartment

Tubular compartment contains germ cells and two different types of somatic cells, the peritubular cells and Sertoli cells. Peritubular cells or myofibroblasts form concentric layers around the tubules separated by collagen layers. They produce several factors involved in cellular contractility such as panactin, desmin, gelsoline, myosin, and actin [54] and secrete extracellular matrix and typical connective tissue cell factors such as collagen, laminin vimentin, fibronectin, growth factors, and adhesion molecules [55,56]. Sertoli cells extend from the basal lamina to the lumen of tubules and envelop and support germ cells during spermatogenesis. This process includes mitotic divisions of spermatogonia that differentiate into spermatocytes and meiotic divisions of spermatocytes to produce spermatids that differentiate into spermatozoa [57]. Several studies investigated GPER role in regulating the several steps of spermatogenesis. In this section, we will summarize the results obtained in studies focused on GPER role in Sertoli and peritubular cells as well as in regulating germ cell maturation (Figure 1).

### 3.1. GPER in Sertoli Cells

Sertoli cells, being intimately associated with each other and germ cells through specific junctions and being primary targets of follicle stimulating hormone [FSH] and testosterone, represent the main mediators of both endocrine and paracrine spermatogenesis control [58]. Considering that each Sertoli cell supports a limited germ cell number, the proliferation of immature Sertoli cells represents an important phenomenon, which determines sperm production capacity. It has been reported that activation of various signaling pathways including cAMP/PKA, ERK1/2, PI3K/Akt, and mTORC1/p70SK6 as well as numerous hormonal factors such as FSH, the insulin family of growth factors, activin, cytokines, and estrogens, are involved in the proliferation of immature Sertoli cells [59]. Particularly, it has been reported that, in fetal and immature rats, Sertoli cells, which are the main source of estrogens [60,61,62], expressed a functional GPER [33] besides ERs [63]. It has been proposed that estrogens regulate Sertoli cell proliferation or Sertoli cell maturation depending on the ER activated. Lucas and colleagues demonstrated that estradiol binding ERα, through MAPK3/1 and PI3K/NFkB pathways, increased CD1 expression and promoted Sertoli cell proliferation, while binding ERβ, through PI3K/CREB signaling, determined an increase expression of CDKN1B cell cycle inhibitor and promoted cell differentiation [64]. Moreover, in immature rat Sertoli cells, estradiol or G1-dependent GPER stimulation, through EGFR transactivation, determined MAPK3/1 phosphorylation that was responsible for proapoptotic BAX decrease and antiapoptotic BCL2 increase in the gene expression [33,65]. Furthermore, E2 or G1 binding GPER upregulated BCL2 and BCL2L2 through the EGFR/MAPK3/1 and PIK3 pathways activation, while through EGFR/MAPK3/1/phospho-CREB signaling, E2 or G1 decreased BAX expression in cultured Sertoli cells from 15 d-old rats [66]. Proliferative effects were also observed in cultured immature boar Sertoli cells; in this cell type GPER triggered a Src/PI3K/Akt pathway activation that was involved in E2-induced cell proliferation via S-phase kinase-associated protein 2 (Skp2) mRNA and protein increase [67]. In another study, it has been demonstrated that proliferation of mouse immature Sertoli cells TM4 can be stimulated by nanomolar concentrations of BPA, through a mechanism involving GPER. Particularly, the authors reported that the rapid activation of GPER/EGFR/ERK1/2 and ERα/β/ERK1/2 pathways was involved in BPA-induced cell growth stimulation. Low doses of BPA significantly increased BCL2 and PCNA expression, while decreased those of p21 and p53. Additionally, BPA up-regulated GPER mRNA and protein expression in a dose-dependent manner, thus contributing to increased stimulatory action of low BPA doses on immature Sertoli cell growth [68]. Taken together, these results suggest that GPER activation in Sertoli cells can modulate the molecular mechanism involved in the maintenance of Sertoli cell number, normal testis development and homeostasis.

### 3.2. GPER in Peritubular Cells

In primates, including humans, testicular peritubular cells are located in a space between the germinal epithelium and the interstitial compartment [69]. Main characteristic of these cells is that they differentiate during puberty by expressing different markers including those of smooth muscle [70]. Several data suggest that estrogens via GPER can regulate both the vascular tone [71] and vascular function [72]. Moreover, it has been demonstrated that human peritubular cells produce several factors such as the GDNF, necessary for spermatogonial stem cell niche [73] and others that play a fundamental role in the spermatogenesis paracrine regulation. Sandner and colleagues [74] demonstrated that GPER is expressed in testicular peritubular cells of human and non-human primates and it is involved in regulation of testicular function. Specifically, in rhesus monkeys these cells express GPER only at the beginning of puberty while in humans this expression is lost due to idiopathic infertility. In tissues deriving from newborn monkeys or until childhood, GPER was mainly expressed in vascular smooth muscle cells; while in those deriving from prepuberal animals, this receptor was only expressed in interstitial cells. In men, the onset of GPER expression in peritubular cells around puberty suggested a role related to fertility. Testicular biopsies from men with normal spermatogenesis showed normal peritubular GPER expression, while those with impaired spermatogenesis exhibited a decreased or deficient GPER immunostaining [74]. These results indicated that estrogenic signaling via GPER is involved in the regulation of smooth muscle-like phenotype of peritubular cells.

### 3.3. GPER in Germ Cells

It is now known that estrogens exert regulatory effects on spermatogenesis through interaction with classic estrogens receptors and also with alternative receptors such as GPER [3,35,75,76,77,78,79,80]. The first report indicating estrogens’ proliferative effects on testicular germ stem cells is attributed to Cobellis and colleagues, who, using a frog model characterized by slow progression of spermatogenesis, demonstrated that estradiol induced nuclear c-Fos activity, leading to spermatogonia multiplication [81]. Other studies corroborated also that estrogens’ proliferative effects on spermatogonia involved ERK1/2 rapid activation [82,83]. Our previous data confirmed GPER expression in mouse testis, supporting the hypothesis that this receptor represents the alternative receptor through which estrogens can sustain spermatogenesis in ERKO mice; in fact, these animals displayed a less severe testicular phenotype compared to that of ArKO mice [78]. Using as experimental model the GC-1, an immortalized cell line that displayed specific features common to type B spermatogonia, we confirmed GPER-dependent pathways involved in spermatogonia cell proliferation. Specifically, we demonstrated that estradiol, through a GPER/ERα cross talk, activated a rapid EGFR/ERK/c-fos signaling cascade, which in turn induced CD1 expression and then cell proliferation [84]. Sheng and colleagues demonstrated that in GC-1 cells low doses of BPA activated the GPER/EGFR/ERK and GPER/PKG pathways, which in turn phosphorylated the transcription factor CREB and the cell cycle regulator Rb. The latter proteins were the key factors involved in the modulation of GC-1 cell proliferation [85]. In another study, the same authors demonstrated that, through the GPER/EGFR/ERK, GPER/EGFR/ERK/ERα/c-Fos, and GPER/PKG pathways, BPA significantly induced GPER gene and protein expression in GC-1 cells. These molecular events contributed to increased cell proliferation via a regulatory loop [86].

The ability of estrogens to induce rapid signaling through GPER and ERs was demonstrated by our research group in primary cultures of pachytene spermatocytes isolated from adult rat testis. In this cell type, physiological concentrations of estradiol as well as G1 and PPT were able to induce rapid ERK1/2 and c-Jun phosphorylation, effects that were abolished by AG1478, PD98059 and ICI182780 inhibitors. Additionally, treatments with E2 or G1 drastically reduced CA1 and B1 mRNA expression. The EGFR and MAPK inhibitors reversed this effect on both cyclins while ER antagonist was able to reverse E2- but not G1-inhibition, thus confirming the E2/GPER/ERK1/2/c-Jun pathway involvement in CA1 and CB1 mRNA expression changes. The inhibition of cyclins expression was associated with an increase of E2 and G1-dependent BAX mRNA expression, which was completely reversed in the presence of EGFR and MAPK inhibitors. Collectively our data indicated that E2, in pachytene spermatocytes, through both ERα and/or GPER, was able to trigger a rapid EGFR/ERK/c-Jun pathway involved in gene expression modulation of cell cycle and apoptosis regulators [31]. In another study, in order to clarify the effector pathways controlling spermatocytes apoptosis, we used the GC-2 [87], an immortalized mouse pachytene spermatocyte-derived cell line. These cells reproduced the same effects that we observed in spermatocytes primary cultures [31]. In fact, in GC-2 cells we demonstrated that ERα and GPER activation by E2, PPT and G1 caused a rapid and sustained ERK and c-Jun phosphorylation, as well as apoptosis. However, only concomitant ERα and GPER silencing abolished E2-dependent effects on cell proliferation. All these data confirmed that E2, by activating both ERα and GPER, was able to decrease cell proliferation through an ERK1/2, c-Jun and p38-dependent mitochondrial apoptotic pathway in GC-2 cells [32]. Using GC-2 cells, Wang and colleagues demonstrated that low doses of BPA, by activating GPER, determined a rapid ERK1/2 phosphorylation, an increase in c-Fos, and a decrease in CD1 gene expression. In addition, BPA-dependent GPER activation inhibited cell growth and induced apoptosis. Moreover, immunohistochemistry analysis, performed on testis of BPA-treated mice, showed that this compound induced spermatocytes apoptosis without affecting the morphological structure of seminiferous tubules [88]. GPER expression was demonstrated also in primary cultures of adult rat RS. In these cells, estradiol, through GPER, ERα and ERβ interaction, activated pathways involved in the regulation of genes controlling rat RS apoptosis and ⁄or maturation. Specifically, E2, PPT, G1 and specific ERβ agonist DPN, caused a rapid ERK1⁄2 phosphorylation increase, through EGFR transactivation. The ability of specific EGFR, ERs and MAPK inhibitors to reverse this effect confirmed the involvement of GPER and ERs in ERK1/2 phosphorylation. Moreover, while E2, G1 and PPT downregulated CB1 and up-regulated BAX mRNA expression, DPN produced the opposite effects on the same genes. All above mentioned results confirmed that E2, through ERs and GPER, modulates rat RS apoptosis and differentiation [30]. Studies investigating the role of GPER in germ cells suggest an important role of this receptor in modulating two important aspects of spermatogenesis: the proliferation of spermatogonia and the physiological apoptosis regulating the number of spermatocytes and spermatids.

## 4. GPER Role in Epididymis

Spermatozoa acquire mobility and fertility during their transit through the epididymis [89]. Epididymal function is regulated by androgens and estrogens signaling. GPER expression was also demonstrated in the epididymis of several species including boar [90], rat [91,92,93], sheep [94] and bull [95], although its immunolocalization by region and cell type is not well known [93]. The highest expression of GPER has been identified in corpus and cauda in adult rats, suggesting a role for non-classical estrogen signaling in sperm maturation in the corpus, and sperm protection/storage in the cauda [93] (Figure 1). Menad and colleagues investigated GPER expression in epididymis of fat sand rats, highlighting how it varied between seasons, among the different segments and across cellular compartments [91]. The same authors also investigated the effects of castration alone or followed by testosterone treatment and efferent ducts ligation. Results evidenced that in castrated animals, GPER underwent a nuclear translocation, while after treatment with testosterone, GPER is mainly localized in the cytoplasm. Instead, in animals with ducts ligation, GPER was mainly localized at the cytoplasmic level in the principal cells of caput epididymis, but with lower intensity in the cauda epididymis [91]. Taken together, these results suggest how GPER can mediate estrogen cell signaling differently between the breeding and resting seasons and how androgens can be involved in regulating GPER expression.

Several studies are available regarding on the presence and actions of ERs in spermatozoa [96,97,98,99,100], but less information exists on GPER. GPER expression was found in the mid-piece, equatorial segment and acrosome of pig spermatozoa, while in humans it was exclusively identified in the sperm mid-piece [101]. This receptor was detected also in the neck, flagellum and head of the stallion spermatozoa [102,103]. In sperm cells isolated from the corpus and caput of the boar epididymis, GPER is less expressed in the acrosome and more expressed in the flagellum, while in epididymal sperm from the cauda, it is mainly localized in the acrosome [90]. This expression pattern suggested a GPER role during the epididymal maturation. It is well known that during this process, spermatozoa undergo several changes leading to their fertilizing ability. These changes include binding of the proteins secreted by epididymis to sperm plasma membrane or sperm proteins post-translational modifications [104]. It is conceivable that proteins expressed between caput and epididymal corpus [105] could bind sperm membrane receptors including GPER. These results suggest a potential involvement of estrogenic signaling in both spermatozoa and epididymis post-testicular maturation. Recently, GPER was revealed in bull ejaculated and capacitated spermatozoa [95], where it was detected in the post-acrosomal region and in the apical part of the acrosome, suggesting its role in rapid signaling such as calcium fluxes [106] and kinase activation [107], which are involved in sperm capacitation and acrosome reaction.

## 5. GPER Role in Testicular Tumors

Testicular tumors account for 1-1.5% of all male cancers [108]; they comprise two broad groups: germ neoplasms (TGCTs) representing 95% of all testicular cancers and the rarer non germ neoplasms which include Leydig cell tumors (LCTs), Sertoli cell tumors and gonadoblastoma.

TGCTs are classified into two sub-categories, based on their histological characteristics, seminoma and non-seminoma (embryonal carcinoma, teratoma, choriocarcinoma and yolk sac tumors), both aroused from a non-invasive form of disease named germ cell neoplasia in situ (CIS) [109]. TGCTs mainly occur in young men (18-35 years old) and their incidence shows geographical and ethnic differences [110]. TGCTs, particularly seminomas, display a good sensitivity to cisplatin-based chemotherapy and radiation; unfortunately, the non-seminoma histotype is more aggressive and has a poor prognosis, since it is less sensitive to chemotherapy and radiotherapy [111,112].

LCTs are a rare disease mainly characterized by interstitial steroids secretion [113] infertility, virilizing syndromes in prepubertal boys, testicular swelling, erectile dysfunction, loss of libido, and gynecomastia [114]. There are two peaks in the LCTs incidence: one during the prepubertal age between 3 and 9 years, the other in adulthood between 30 and 60 years [114]. LCTs are usually benign while an estimated 10–15% are malignant [115]. Mainly treatment of LCTs is the surgical resection for both benign and malignant pathological forms. However, therapeutic options are very limited and include the use of radiotherapy and chemotherapy often ineffective [116]. These therapeutic approaches are complicated by the elevated testicular susceptibility to their toxic effects [117]. Since the prognosis is poor with an average survival of approximately two years [115,116,118], it is necessary identify new therapeutic approaches for the treatment of malignant LCTs.

**Figure 2:**
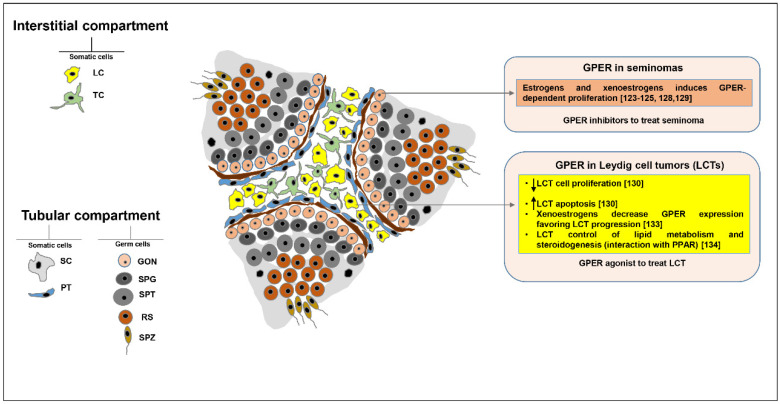
GPER role in testicular tumorigenesis. In this figure are summarized the knowledge ofGPER role in seminomas and Leydig cell tumors [LCTs]. LC: Leydig cells; TC: telocytes; SC: Sertoli cells; PT: peritubular cells; GON: gonocytes; SPG: spermatogonia; SPT: spermatocytes; RS: round spermatids; SPZ: spermatozoa.

It has been shown that GPER is expressed in TGCTs. Specifically, it is overexpressed in seminoma and embryonal carcinoma [119], whereas ERα is missing. It suggests that in these tumors estrogens can exert proliferative effects through GPER (Figure 2).

Rago and colleagues demonstrated that seminomas and embryonal carcinomas had a positive ERβ1 and ERβ2 immunoreactivity, while ERα signal was undetectable [120]. Indeed, in a seminoma cell line which lacks ERα expression, ERβ activation has been shown to be associated with cell necrosis and autophagy [121]. Using the JKT-1, a cell line derived from a human testicular seminoma and expressing GPER, ERβ, but not ERα [122], it has been demonstrated that estradiol [123], BPA [124,125] and G1 through GPER activated a PKA/CREB signaling leading to cell proliferation increase. On the other hand, E2 through ERβ [126,127] or G15 through GPER/ERK pathway, decreased cell proliferation. In another study, it has been demonstrated that estradiol through a GPER-cAMP/PKA/CREB signaling induced ERα36 isoform expression in TCam-2 seminoma cell line, leading to cell proliferation increase [128]. Boscia and colleagues showed that GPER overexpression was associated with ERβ down regulation in both human testicular CIS and seminomas. In addition, ERβ reduced expression was due to the GPER/ERK/c-Fos estrogen activated pathway in TCam-2 seminoma cell line. The observation that high levels of GPER protein correlated with low levels of ERβ suggested a potential therapeutic role of GPER inhibitors for the treatment of CIS and seminomas [129] (Figure 2).

On the contrary, in Leydig cell tumors, where ERα is overexpressed, GPER activation is associated with a marked reduction of cell growth in vitro and in vivo [130] (Figure 2). Specifically, G1 treatment determined inhibitory effects consequent to the initiation of the mitochondria-dependent apoptotic pathway in R2C Leydig tumor cell line [130]. This pathway that required a sustained ERK1/2 activation, was confirmed by DNA fragmentation, a decrease in BCL2 and an increase in BAX expression, cytochrome c release, caspase 3, and PARP1 activation after G1 treatment. These effects have been demonstrated for other tumor cell types including those of the breast [131] and prostate [132]. In addition, in vivo administration of G1 to male CD1 mice, decreased the growth of R2C xenograft tumors without any alteration in testicular morphology [130]. All results suggested that GPER may represent a potential therapeutic option to preserve fertility for Leydig cell tumor treatment [130].

An innovative aspect in the study of GPER role in Leydig cell tumors is the functional interaction among GPER and other receptors. Recently, Gorowska-Wojtowicz and colleagues [133] examined mainly the relationship between GPER and PPARs expression and estrogen level after several xenoestrogens treatment. The authors demonstrated that in tissues from mice MA-10 xenograft models and in MA-10 cells treated with BPA and TCBPA, PPARα expression was increased while that of GPER, PPARβ and PPARγ was decreased together with estradiol production. These results indicated that changes in GPER and PPARs expression after xenoestrogens exposure observed both in vivo and in vitro are involved in the alteration of cell tumor microenvironment. Recently, Kotula–Balak and colleagues highlighted that the functional interaction between GPER and PPAR controls lipid metabolism and steroidogenesis in LCTs. In fact, in these tumors, analysis of mRNA and proteins showed a greater expression of GPER and a reduction of PPARα, β and γ expression. Consequently, it was also observed that there are alterations in lipid- and cholesterol-associated proteins such as LHR, PKA, PLIN, HSL, StAR, TSPO, HMGCS, and HMGCR, together with the cGMP and PI3K-Akt-mTOR pathways. These changes of expression could be primary disturbances in healthy Leydig cell, and the study of these alterations could be useful to design a new therapeutic approach for Leydig cell tumors [134].

Another interesting aspect that could be investigated in the future for the treatment of testicular tumors, arises from the observation that GPER activation has combinatorial effects with immune checkpoint inhibitors. In fact, in a recent paper, Natale and colleagues demonstrated that GPER activation made tumors more responsive to immune checkpoint blockade increasing survival, with up to half of mice clearing their tumor [135]. Very recently, the same research group observed similar results on Pancreatic Ductal Adenocarcinoma [PDAC] [136]. In particular, G1 treatment inhibited PDAC proliferation, depleted the oncodriver and stem cell marker c-Myc, depleted PD-L1, and increased tumor cell immunogenicity. These observations on preclinical models are supported by the preliminary results obtained by clinical trial on patients with advanced solid and hematologic cancers treated with LNS8891, an highly specific GPER agonist [137].

Taken together, these results suggest GPER as a useful potential target for the development of new pharmacological tools against testicular tumors. 

## 6. Conclusions

The discovery of the transmembrane estrogen-binding protein GPER has opened new perspectives to better understand molecular mechanisms mediating estrogen action in testicular physiology, as well as in testicular tumors (Table 1).

In interstitial compartment, GPER appears to play an important role in regulating estrogen-dependent lipid homeostasis and testosterone biosynthesis that occur in Leydig cells. Furthermore, very recent results have also shown an important role of GPER in regulating the number and physiology of telocytes that contribute to maintain lipid balance and steroidogenic efficiency of interstitial tissue. In tubular compartment, GPER can mediate estrogen action on both somatic as well as germ cells. The reduced GPER expression in peritubular cell seems to be associated to infertility, while the role of GPER in the maintenance of Sertoli cell number and consequently for normal testis development and homeostasis is well known. Studies investigating GPER action in germ cells suggest an important role of this receptor in modulating two important aspects of spermatogenesis: the proliferation of spermatogonia and the physiological apoptosis regulating spermatocytes and spermatids number. GPER seems to be also involved in mediating signaling regulating spermatozoa maturation in epididymis.

An interesting aspect discovered in the last years is the functional interaction among GPER and other receptors such as ERs, PPARs, and ERRs in interstitial and tubular compartments. This aspect has recently opened a new interesting perspective in understanding the lipid homeostasis and metabolism of testis. This last aspect is particularly important for the study of testicular tumorigenesis. In fact, in Leydig cell tumors, greater expression of GPER and a reduction of the PPARα, β and γ expression are correlated with the alterations in several lipid- and cholesterol-associated proteins, determining disturbances that could be related to tumorigenesis and cancer progression. Moreover, results indicating that GPER activation by selective ligands can allow for opposite outcomes in seminoma and LCTs (Figure 2) should open new perspectives to define the mechanisms behind estrogen-dependent testicular tumorigenesis. In this context, GPER appears to be a very interesting potential target together with other nuclear receptors for the development of new pharmacological tools against estrogen-dependent testicular cancer.

## Figures and Tables

**Table 1:** Participation of GPER in testicular physiology in main cell types and changes in testicular tumors.

Cell Types	Testicular Physiology	Testicular Tumors
SPG	GPER activation induces proliferation [84,85,86]	GPER is overexpressed in seminoma and embryonal carcinoma [119], (whereas ERα is missing [120]) GPER mediates estrogen and xenoestrogen-dependent proliferation in seminomas [123,124,125,128,129]
SPT	GPER activation induces apoptosis [31,32,88]	
RS	GPER activation induces apoptosis [30]	
LC	GPER is involved in: LC morphology and function [38]	LCT [where ERα is overexpressed]: GPER activation decrease cell proliferation and induces apoptosis [130] Xenoestrogens decrease GPER expression favoring LCT progression [133] GPER is involved in LCT control of lipid metabolism and steroidogenesis (interaction with PPAR) [134]
	LC androgens production [40]	
	LC lipophagy inhibition [43]	
	LC miRNA biogenesis [49]	
	LC development and function [PPAR crosstalk] [46]	
SC	GPER activation induces proliferation in immature testis [65,66,67,68]	Sertoli cell tumors: GPER is expressed but the role has been not identified.

SPG: spermatogonia; SPT: spermatocytes; RS: round spermatids; SC: Sertoli cells; LC: Leydig cells; Leydig cell tumor [LCT].

## Abbreviations

AC	adenylyl cyclase;
AGO2	argonaute 2;
Akt	protein kinase b [PKB];
ArKO	aromatase knockout;
BAX	BCL2 associated X protein;
BCL2	B-cell lymphoma 2;
BCL2L2	BCL2 like protein 2;
BPA	bisphenol a;
cAMP	cyclic adenosine monophosphate;
CDKN1B	cyclin dependent kinase inhibitor 1b;
CA1	cyclin A1;
CB1	cyclin B1;
CD1	cyclin D1;
cGMP	cyclic guanosine monophosphate;
CIS	germ cell neoplasia in situ;
CREB	cAMP response element-binding protein;
DICER	dicer 1, ribonuclease III;
DPN	2,3-bis[4-Hydroxyphenyl]-propionitrile;
DROSHA	drosha ribonuclease III;
E2	17beta-estradiol;
EGFR	epidermal growth factor receptor;
ERα	estrogen receptor α;
ERβ	estrogen receptor β;
ERs	estrogen receptors;
ERE	estrogen response element;
ERK	extracellular signal-regulated kinase;
ERKO	estrogen receptor knockout;
ERRβ	estrogen-related receptor β;
ERRs	estrogen-related receptors;
EXPO5	exportin-5;
FSH	follicle stimulatimg hormone;
G15	[3aS,4R,9bR]-4-[6-bromo-1,3-benzodioxol-5-yl]-3a,4,5,9b-tetrahydro-3H-cyclopenta[c]quinolone;
G1	[±]-1-[[3aR*,4S*,9bS*]-4-[6-Bromo-1,3-benzodioxol-5-yl]-3a,4,5,9b-tetrahydro-3H-cyclopenta[c]quinolin-8-yl]-ethanone;
GDNF	glial cell-derived neurotrophic factor;
GPCR	G protein-coupled receptor;
GPER	G protein-coupled estrogen receptor 1;
HMGCR	hydroxymethylglutaril-coa reductase;
HMGCS	hydroxymethylglutaril-coa synthase;
3β-HSD	3β-hydroxysteroid dehydrogenase;
HSL	hormone sensitive lipase;
LC3	microtubule-associated protein 1A/1B-light chain;
LCT	leydig cell tumor;
LHR	luteotropin receptor;
MAPK	mitogen-activated protein kinase;
mTOR	mammalian target of rapamycin;
mTORC1	mammalian target of rapamycin complex 1;
NFkB	nuclear factor kappa-light-chain-enhancer of activated B cells;
p70SK6	ribosomal protein s6 kinase beta-1;
PARP1	poly [ADP-ribose] polymerase 1;
PCNA	proliferating cell nuclear antigen;
PDAC	pancreatic ductal adenocarcinoma;
PDGFR	platelet-derived growth factor receptor;
PD-L1	programmed death ligand 1;
PI3K	phosphoinositide 3-kinase;
PKA	protein kinase a;
PKG	cGMP-dependent protein kinase;
PLC	phospholipase c;
PLIN	perilipin;
PPARα	peroxisome proliferator-activated receptor α;
PPARβ	peroxisome proliferator-activated receptor β;
PPARγ	peroxisome proliferator-activated receptor γ;
PPARs	peroxisome proliferator-activated receptors;
PPT	4,4′,4″-[4-Propyl-[1H] -pyrazole-1,3,5-triyl] trisphenol;
Rb	retinoblastoma protein;
RS	round spermatids;
Skp2	s-phase kinase-associated protein 2;
Src	proto-oncogene tyrosine-protein kinase;
StAR	acute steroidogenic regulatory protein;
TBBPA	tetrabromobisphenol A;
TCBPA	tetrachlorobisphenol A;
TGTCs	testicular germ cell tumors;
TORC1	mammalian target of rapamycin complex 1;
TSPO	translocator protein;
VEGF	vascular endothelium growth factor;
